# Regulation of Expression of Citrate Synthase by the Retinoic Acid Receptor-Related Orphan Receptor α (RORα)

**DOI:** 10.1371/journal.pone.0033804

**Published:** 2012-04-02

**Authors:** Christine Crumbley, Yongjun Wang, Subhashis Banerjee, Thomas P. Burris

**Affiliations:** Department of Molecular Therapeutics and Center for Diabetes and Metabolic Disease, The Scripps Research Institute, Jupiter, Florida, United States of America; Baylor College of Medicine, United States of America

## Abstract

The retinoic acid receptor-related orphan receptor α (RORα) is a member of the nuclear receptor superfamily of transcription factors that plays an important role in regulation of the circadian rhythm and metabolism. Mice lacking a functional RORα display a range of metabolic abnormalities including decreased serum cholesterol and plasma triglycerides. Citrate synthase (CS) is a key enzyme of the citric acid cycle that provides energy for cellular function. Additionally, CS plays a critical role in providing citrate derived acetyl-CoA for lipogenesis and cholesterologenesis. Here, we identified a functional RORα response element (RORE) in the promoter of the CS gene. ChIP analysis demonstrates RORα occupancy of the CS promoter and a putative RORE binds to RORα effectively in an electrophoretic mobility shift assay and confers RORα responsiveness to a reporter gene in a cotransfection assay. We also observed a decrease in CS gene expression and CS enzymatic activity in the staggerer mouse, which has a mutation of in the *Rora* gene resulting in nonfunctional RORα protein. Furthermore, we found that SR1001 a RORα inverse agonist eliminated the circadian pattern of expression of CS mRNA in mice. These data suggest that CS is a direct RORα target gene and one mechanism by which RORα regulates lipid metabolism is via regulation of CS expression.

## Introduction

The Retinoic Acid Receptor-Related Orphan Receptors (RORs) belong to the nuclear hormone receptor superfamily. Like all nuclear hormone receptors, the RORs possess a canonical domain structure composed of a unique N-terminal region, a highly conserved DNA binding domain of two zinc fingers, a hinge region, and a ligand binding domain that binds to ligands and interacts with transcriptional coregulatory proteins. RORα has been shown to bind to cholesterol and cholesterol sulfate [1], [2], and our recent work has demonstrated that 7-oxysterols as well as other oxysterols bind to RORα with high affinity (<50 nM Ki) and suppress their transcriptional activity [3], [4]. Most recently, we have identified both synthetic agonists and inverse agonists that target RORα that we have begun to use as chemical probes to understand RORα function [5], [6], [7], [8], [9].

RORα is well known for its role in regulation of the circadian rhythm, given that core clock components such as *BMAL1* and *NPAS2* are direct target genes of RORα [10]. However, RORα also plays an important role in regulation of metabolism [10]. Several studies have identified genes important in regulation of lipid and glucose metabolism as RORα target genes including *apolipoprotein A1* [11], *Cyp2C8* [12], *Cyp7b1* [13] and *glucose 6-phosphatase* [14]. *Staggerer* (*sg/sg*) mice that have an inactivating mutation in the *Rora* gene display metabolic abnormalities including decreased serum cholesterol [15] and plasma triglycerides [16]. Additionally, *sg/sg* mice display lower levels of hepatic expression of *Srebp-1c* and are resistant to diet induced obesity [17].

Citrate synthase (CS) catalyzes the first step of the citric acid cycle. Oxaloacetate and acetyl-CoA produced from pyruvate are converted to citrate by CS. This citrate can then continue in the citric acid cycle to produce ATP for the cell or it can be transported to the cytosol, where it is converted back to acetyl-CoA. In the cytosol, this citrate-derived acetyl-CoA is converted to malonyl-CoA by acetyl-CoA carboxylase, which is the committed step in lipid synthesis, or to acetoacetyl-CoA, which is a step in the cholesterol synthesis pathway. Here, we describe our studies where we have found that this enzyme that plays a critical role in energy production and lipid biosynthesis is regulated by RORα.

## Methods

### Reagents

The RORα pTREX vectors were a gift from Phenex Pharmaceuticals AG (Ludwigshafen, Germany). The RORα pcDNA3.1+ constructs were generated by amplifying the ROR sequence from ROR-pSport6 vectors, digesting the products with HindIII and BamHI (Promega), gel purifying (Qiagen), and ligating overnight at room temperature using T4 DNA ligase (Promega). The ROR-pSport6 vectors were a gift from the Cell-based Screening Center at The Scripps Research Institute ( Jupiter, FL). The CS luciferase reporter construct was generated by amplifying a fragment of the CS promoter using primers designed Kraft *et al* [18]. The mutant CS reporter was generated by deleting two nucleotides from the ROR response element using the QuikChange XL kit, according to the manufacturer’s instructions (Stratagene). All constructs were confirmed by sequencing. The adenoviral vector for RORa was described previously [5]. 

### Cell Culture and Transfection

HepG2 cells (ATCC, Manassas VA) were maintained in minimal essential medium with 10% fetal bovine serum at 37C with 5% CO_2_. 24 hours prior to transfection, HepG2 cells were plated at a density of 15×10^3^ cells/well in a 96-well plate. Lipofectamine 2000 (Invitrogen) was used as the transfection reagent. Per well, transfection mixtures contained 50 ng of the Renilla luciferase (internal control), 100 ng of the appropriate CS::Luciferase construct, and 100 ng of the appropriate ROR-pTREX expression construct. 24 hours after transfections, cells were harvested to determine luciferase activity, which was measured using the Dual-Glo assay system (Promega). During analysis, each luciferase reading was normalized by well to the Renilla readings.

### ChIP/chip Screening

HepG2 cells were infected with RORα adenovirus and harvested for ChIP/chip screening as previously described [5].

### ChIP

HepG2 cells were cultured in a 10 cm dish until ∼80% confluency. Cells were fixed using 37% paraformaldehyde (Sigma). The ChIP-It Express kit (Active Motif) was used to perform the ChIP assay. Cells were lysed and sonicated. Immunoprecipitations were incubated at 4C overnight. The ChIP reactions contained the following antibodies: IgG (Active Motif), anti-RNA Pol II (Active Motif), anti-hRORα (Santa Cruz Biotechnology, sr-6062X). The ChIP reactions also contained salmon sperm DNA (2.5 ug/uL) and BSA (2.5 ug/uL). ChIP reactions were washed and chromatin was eluted, according to manufacturer’s instructions. Chromatin was purified using PCR clean up columns (Qiagen). PCRs were performed using Supermix High Fidelity (Invitrogen), 1.5 uL of each primer (10 uM), and 10 uL of chromatin. The CS primer sequences are CS_RORE1_ChIP_F: CTCCAGAGGAGCACTGACCT and CS_RORE1_ChIP_R: ACCCTGTCGAGAGGCTTAGA. PCR products were analyzed using ethidium bromide staining and electrophoresis.

### Electrophoretic Mobility Shift Assay (EMSA)

The RORα pcDNA3.1+ constructs contain a T7 promoter that allows for transcription and translation to occur in vitro. Protein was produced using the TNT T7 Quick Coupled kit (Promega), according to manufacturer’s instructions. DNA containing putative ROREs was annealed, labeled with T4 Polynucleotide Kinase (Promega) and γ-P32-ATP (Perkin Elmer Life and Analytical Sciences), and purified using Sepharose columns (Roche). Binding reactions contained binding buffer (Promega), labeled probes, and protein. For competition experiments, unlabeled probes were used at 10-, 50-, and 100-fold molar excess. Binding reactions were loaded onto pre-cast 5% TBE gels (Biorad) and analyzed by autoradiography.

### mRNA Extraction, cDNA Synthesis, and Quantitative PCR

The mRNA extraction, cDNA synthesis, and quantitative PCR were performed as described previously [19]. For qPCR, the cyclophilin B (M60857) served as the control gene. All primers were designed for human genes. The sequences of qPCR primers are: hCycB_F: 5′-GGAGATGGCACAGGAGGAAA-3′, hCyCB_R:5′-CGTAGTGCTTCAGTTTGAAGTTCTCA-3′, hCS_F:5′-TAGTGCTTCCTCCACGAATTTG-3′, and hCS_R: 5′-CCACCATACATCATGTCCACAG-3′.

### Adenovirus Infection

HepG2 cells were plated 24 hours prior to infection. Adenovirus was added to the cells for 24 hours and then media was changed to fresh MEM media. The cells were harvested 24 hours after media change.

### CS Enzymatic Activity Assays

HepG2 cells were cultured then infected with adenovirus. HepG2 cells were lysed with CelLytic M buffer with added protease inhibitor (1∶100 concentration, Sigma). Total protein concentration was determined using a Bradford assay. The assay contained 40 µg total protein per well (120 µg used in the master mix, which was aliquoted into three wells). Enzymatic activity was determined using the CS assay kit (Sigma). The A412 readings were taken on a Spectramax5 plate reader (Molecular Devices) with 7 readings over the 1.5 min time span. These readings were in the linear range of enzymatic activity. The difference between baseline and OAA-treated samples was obtained and used to calculate total CS activity according to the formula provided in the manual.

### Mouse Experiments

The livers from *staggerer* mice, a naturally occurring RORα mutant were purchased from Jackson laboratories. Liver was homogenized on ice. mRNA was prepared by the Trizol method. For the qPCR, cyclophilin B was the control gene. qPCR was performed using the following primers: mCycB_F: 5′-GCAAGTTCCATCGTGTCATCAAG-3′, mCycB_R: 5′-CCATAGATGCTCTTTCCTCCTG-3′, mCS_F: 5′-GGACAATTTTCCAACCAATCTGC-3′, and mCS_R: 5′-TCGGTTCATTCCCTCTGCATA-3′. For CS assays liver was homogenized on ice using CelLytic MT buffer with added protease inhibitor (1∶100 concentration, Sigma). Protein quantity was determined using a Bradford assay. The assay contained 4 µg protein per well (12 µg total in the master mix, which was aliquoted into 3 wells). Enzymatic activity was determined using the CS assay kit, as described above. For circadian gene expression experiments male C57BL6 mice (8–10 weeks of age) were either maintained on a L:D (12h∶12h) cycle or on constant darkness (1 day). At circadian time (CT) 0 animals were administered a single dose of 25 mg/kg SR1001 (i.p.) and groups of animals (n = 6) were sacrificed at CT0, CT6, CT12 and CT18. Gene expression was determined by real time QPCR. Gene expression was normalized to Cyclophin b in all experiments. These studies were carried out in strict accordance with the recommendations in the Guide for the Care and Use of Laboratory Animals of the National Institutes of Health. The protocol was approved by the Institutional Animal Care and Use Committee of The Scripps Research Institute.

### Statistical Analysis

In the co-transfection assays, 8 wells were transfected per condition per experiment. For gene expression assays, 3 to 4 experimental replicates and 3 technical replicates were used. Experiments were repeated 3 times and data are shown as mean±SE. Student’s t-test was used to evaluate potential significant differences between the groups.

## Results

**Figure 1:**
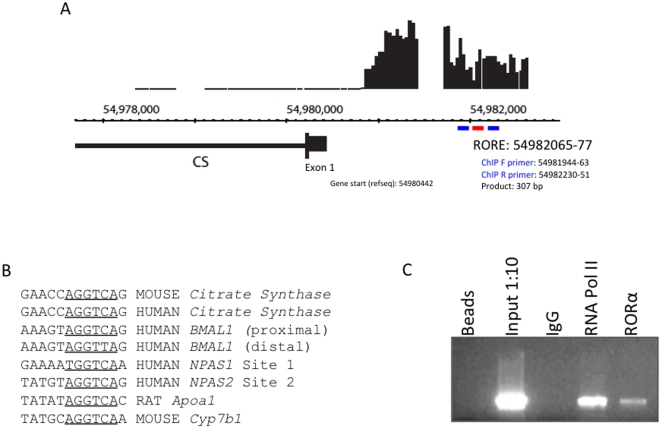
Identification of Citrate Synthase as a Putative RORα Target Gene. A. Screen shot from Genome Browser illustrating the RORα binding signal. Using HepG2 cells over-expressing RORα using adenovirus, a ChIP-on-chip screen identified a region of RORα binding in the hCS promoter. The hCS gene is located on chromosome 12. Positive signal intensity reflecting RORα occupancy is indicated on the top by the vertical lines. Horizontal lines just below the signal intensity refer to areas of chip coverage. Position within the chromosome is indicated by the numbered scale. On the bottom, the gene structure is illustrated showing the first exon and first intron of the CS gene. RORα binding was detected in the promoter in ∼2.5 kb region upstream of the first exon. ChIP primers used for confirmation of binding to the promoter are indicated in blue. The putative RORE is indicated in red. B. Conserved RORE sequence was identified in the human and mouse CS promoter on the Evolutionarily Conserved Region Browser. The putative RORE is positioned at 54,982,065–54,982,077; whereas the start site is positioned at ∼54,980,442. C. Endogenous levels of RORα in HepG2 cells can be detected at the hCS promoter region containing the identified putative RORE. The beads and IgG serve as negative controls, whereas the input and RNA Pol II serve as positive controls.

A ChIP/chip screen was performed as previously described to identify regions within the genome bound by RORα [19]. We observed significant RORα occupancy in the *CS* promoter region (Fig. 1A). The CS promoter was examined using the Evolutionarily Conserved Region Browser (TRANSFAC professional V10.2 library) [20], revealing a consensus ROR response element (RORE) ∼1.5 kb upstream that is absolutely conserved between mice and humans (Fig. 1B). The putative CS RORE aligns well with other ROREs from RORα target genes (Fig. 1B). We confirmed our ChIP/chip data using a standard ChIP assay and as illustrated in Fig. 1C, we observed strong RORα occupancy of the *CS* promoter.

**Figure 2:**
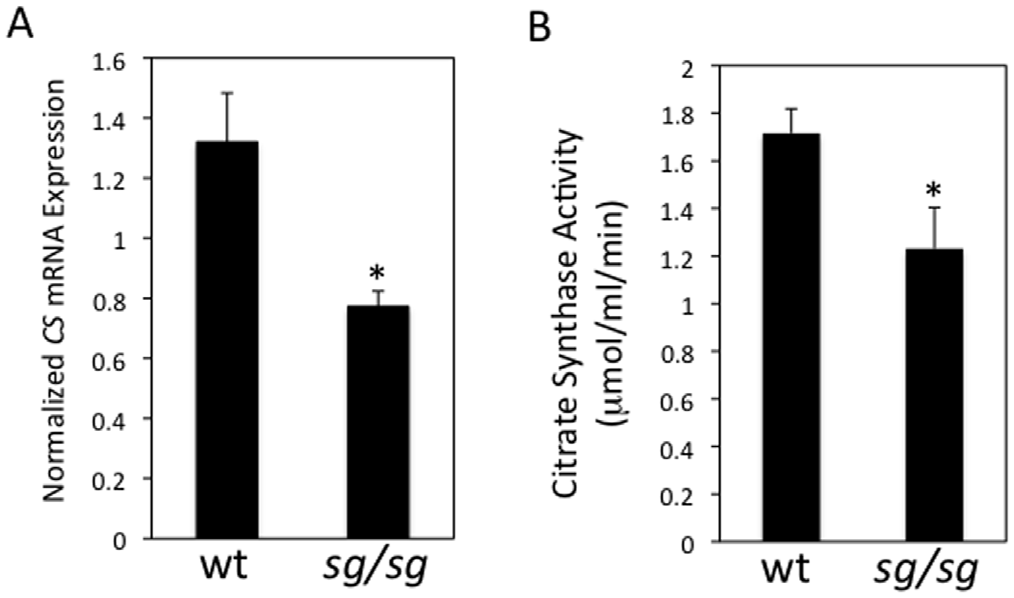
Citrate Synthase Expression and Enzyme Activity is Suppressed in RORα-Deficient *Staggerer* Mice. A. The mRNA expression of *CS* in liver is reduced by 42% in the *staggerer* mice relative to the wild-type mice. The mRNA expression was determined using RT-PCR and normalized to cyclophilin B expression. B. CS enzyme activity in *sg/sg* mice vs. wt mice. Liver was homogenized and the protein extract was used to perform a colorimetric assay to determine CS activity. The activity of the CS protein is reduced by 29% in the *staggerer* mice relative to the wild-type mice. Data are shown as mean ± standard error (n = 4); an asterisk (*) indicates a p-value of <0.05.

We sought to determine if RORα played an important role in regulation of CS gene expression and activity by first examining the CS in *staggerer (sg/sg)* mice that harbor a mutant *Rora* gene that renders the RORα protein inactive. We first examined the expression of *CS* gene in wt mice and compared the levels to *sg/sg* mice. In the *sg/sg* mice, *CS* mRNA expression in liver was decreased by 42% compared to wild-type liver (Fig. 2A). This correlated with reduced enzymatic activity, as the CS enzymatic activity was 29% lower in the *sg/sg* liver compared to the wild-type liver (Fig. 2B). These data suggest that RORα does indeed play a role in regulation of *CS* expression and along with the ChIP data, indicates that *CS* is likely a direct RORα target gene.

**Figure 3:**
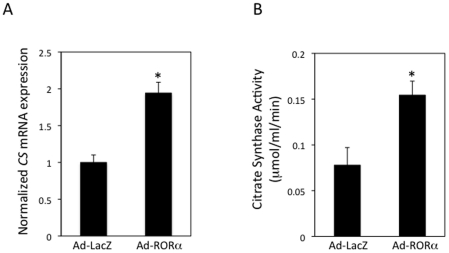
Overexpression of RORα Leads to Elevated *CS* gene expression and CS Enzyme Activity. A. *CS* mRNA expression in HepG2 cells is increased by 94% in the cells infected with RORα adenovirus relative to cells infected with LacZ adenovirus. mRNA expression was determined using RT-PCR and normalized to cyclophilin B expression. B. CS enzymatic activity is increase in HepG2 cells overexpressing RORα. HepG2 cells were homogenized and the protein extract was used to perform a colorimetric assay to determine CS activity. The activity of the CS protein is increased by 98% in the cells infected with RORα adenovirus relative to cells infected with LacZ adenovirus.

After examining CS expression and activity in a RORα loss-of-function model, we performed the converse experiment with a RORα gain-of-function model where we overexpressed RORα in HepG2 cells. We used an adenovirus encoding RORα to elevate the levels of RORα. Forty-eight hours after infection, mRNA was isolated and *CS* gene expression was examined. Overexpression of RORα (5-fold, data not shown), led to a 94% increase in *CS* mRNA levels (Fig. 3A). We also assessed CS enzyme activity in HepG2 cells infected with the RORα adenovirus and observed a 98% increase in enzyme activity relative to HepG2 cells infected with control LacZ adenovirus (Fig. 3B). 

**Figure 4:**
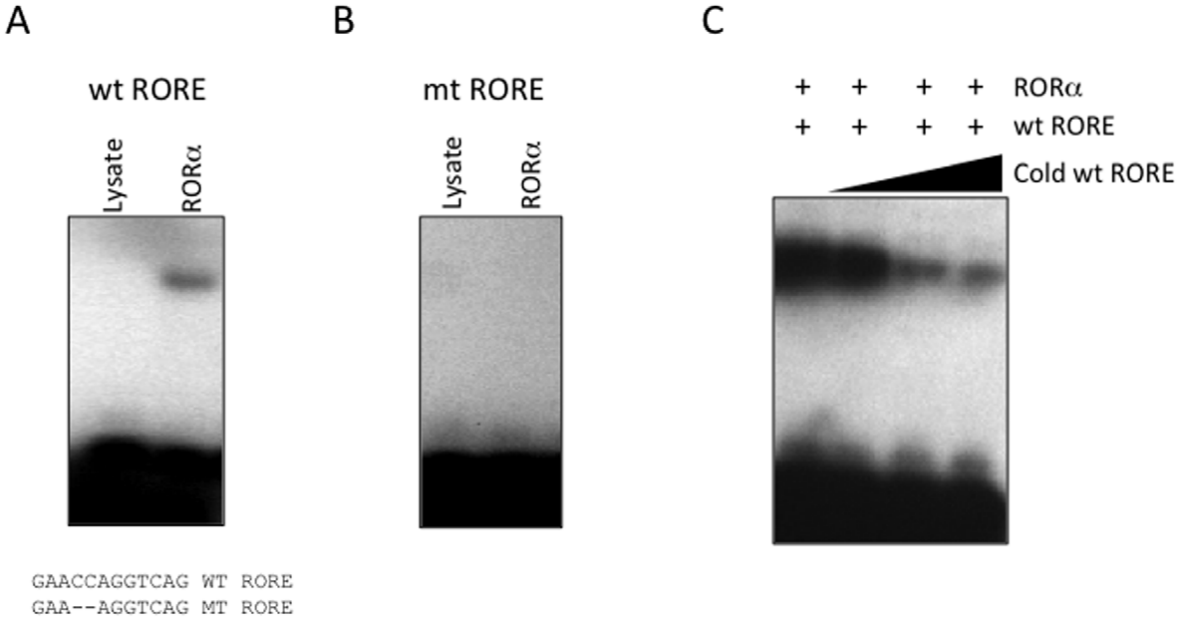
*In vitro* characterization of RORα Binding to the Putative *CS* RORE. Using an EMSA assay, it is determined that RORα binding to the CS RORE is specific and competitive. A. The blank lysate does not contain any proteins that are capable of binding to the radiolabeled RORE sequence. When a protein expression construct encoding RORα was added to the lysate, the produced RORα protein is capable of binding to the radiolabeled RORE sequence. The wt RORE DNA probe and the mutant DNA probe are indicated. Note that the mutant probe has a 2 bp deletion within the 5′ half site extension that is required for ROR recognition of its half site. B. The binding of the radiolabeled RORE is specific for RORα. When a mutant RORE containing a 2 bp deletion was incubated with both blank and RORα lysates, no binding was detected with either sample. C. The amount of RORα protein and radiolabeled RORE were held constant, but the concentration of unlabeled RORE was increased. As the concentration of the unlabeled RORE was increased from 0x to 100-fold molar excess, the binding of the radiolabeled RORE was reduced due to competition from the unlabeled RORE.

Our data suggests that the CS gene is a direct target gene of RORα thus we sought to identify the RORE within the promoter that confers RORα responsiveness. We identified a putative RORE that was absolutely conserved between the mouse and human genes using the Evolutionary Conserved Region Browser [20] (Fig. 1B). We evaluated that ability of this putative RORE sequence to bind to *in vitro* translated RORα using an EMSA. Clearly RORα was able to bind to the radiolabeled CS RORE as shown in Fig. 4A. We examined the specificity of binding of RORα to the RORE by comparing binding with a mutated RORE where we eliminating 2 nucleotides within the core RORE sequence. RORα failed to bind to the mt RORE (Fig. 4B). Furthermore, we observed competitive binding with unlabeled wt RORE probe as would be expected. When the unlabled wt RORE was added to the binding reaction at 10-, 50-, and 100-fold molar excess, the labeled RORE probe was displaced from the binding complex (Fig. 4C).

**Figure 5:**
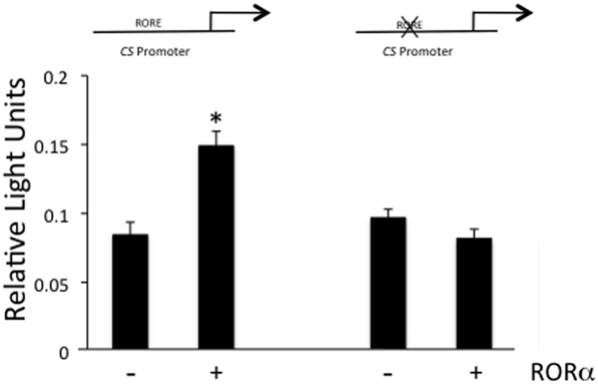
The Citrate Synthase RORE is Functional in a Cotransfection Assay. Cotransfection assays in HepG2 cells demonstrate that expression of RORα can stimulate luciferase gene expression from the *CS::luc* construct. When the *CS::luc* construct contains a 2 bp deletion in the RORE, co-transfection of RORα does not stimulate luciferase gene expression. Data are shown as mean ± standard error (n = 8); an asterisk (*) indicates a p-value of <0.05. The mutant RORE was identical to that used in Fig. 4.

To further examine the direct regulation of the *CS* gene by RORα, we transfected a luciferase reporter gene driven by the *CS* promoter in HepG2 cells. The reporter construct was composed of a ∼1 kb fragment of the CS promoter that contains the putative RORE. We also constructed a similar reporter construct that was identical except for mutation of the RORE as described in the EMSA experiment. As shown in Fig. 5, expression of RORα along with the wt *CS::luc* reporter yielded a 76% increase in reporter transcription while the mt *CS::luc* reporter was unresponsive to RORα confirming that this RORE is a functional RORE within the CS promoter.

**Figure 6:**
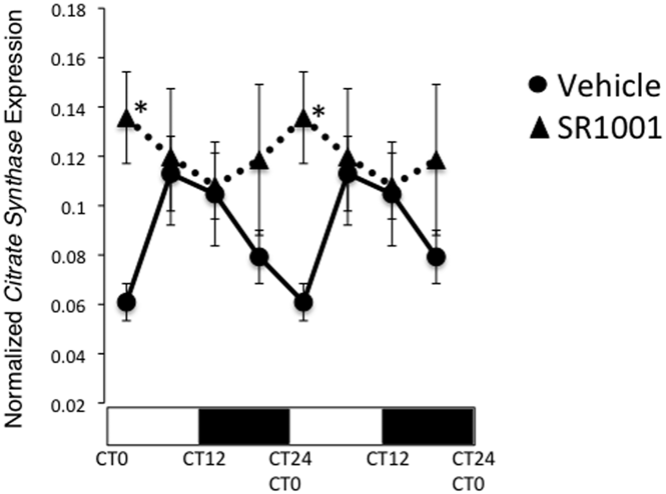
RORα plays an important role in the circadian pattern of expression of citrate synthase mRNA in the liver. Mice were treated with either vehicle or SR1001 at CT0and groups of mice were sacrificed at CT0/24, CT6, CT12 and CT18 followed by assessment of CS gene expression by QPCR. The light/dark bar on the bottom of the graph reflects daytime vs. nighttime. Data has been double plotted for clarity. An asterisk (*) indicates a p-value of <0.05.

Metabolic processes are tightly coupled to the circadian clock and the expression of many genes involved in carbohydrate and lipid metabolism follow a circadian pattern of expression [21], [22]. In fact, the enzymatic activity of CS has previously been reported to display a circadian rhythm [23]. As illustrated in Fig. 6, we also observed a circadian pattern of expression of *CS* mRNA in mice. When these mice were injected with SR1001, an inhibitor of RORα activity the circadian rhythm of *CS* expression was eliminated suggesting that RORα plays an important role in regulation of the circadian pattern of expression of this gene.

## Discussion

Our study demonstrates that the CS gene is a direct target gene of RORα. We identified CS as a putative target gene using a ChIP/chip screen and confirmed RORα occupancy of the promoter by ChIP. Futhermore, RORα appears to play an important role in regulation of CS *in vivo* since mice that lack functional RORα display reduced hepatic *CS* gene expression and CS enzyme activity. Our results also identify a functional RORE within the *CS* promoter that is absolutely conserved between the mouse and human genes.

CS plays an essential role in lipogenesis and cholesterologenesis where citrate produced by CS is transported to the cytosol through the pyruvate-citrate shuttle. Cytosolic citrate is converted to acetyl-CoA by ATP citrate lyase and this reaction is the source of cytosolic acetyl-CoA that is used for cholesterol and lipid biosynthesis. Our observation that CS is regulated by RORα is interesting given the phenotype of the *staggerer* mice with respect to their plasma lipid levels. Both cholesterol and triglyceride levels are reduced in these mice [15], [16], which is consistent with CS levels being reduced as we have observed. Thus, RORα regulation of CS expression may be one mechanism by which RORα regulates lipid and cholesterol homeostasis. Many genes encoding metabolic enzymes are regulated in a circadian fashion and provide a link between circadian behavior and circadian regulation of metabolic processes. CS enzyme activity in the rat follows a circadian pattern with elevated levels of activity during the dark in both the heart and liver [23]. We observed a similar pattern of expression of CS mRNA and found that blocking RORα action with the synthetic ligand SR1001 [8] eliminated the rhythmicity of expression of the mRNA in the liver suggesting that RORα plays a critical role in maintenance of this rhythm. RORα, of course, is well characterized for its role in regulation of the circadian clock and expression of an array of genes involved in carbohydrate and lipid metabolism [10] [19], [24], [25], [26], [27] and thus appears to be one link by which circadian regulation of these genes may be controlled. CS also plays an essential role in energy production and the implications of RORα regulation of the citric acid cycle is less clear at this point.

RORα regulates myriad metabolic pathways and based on the phenotype of the *staggerer* mice as well as the RORα null mice, RORα is a potential target to treat diseases such as atherosclerosis, diabetes, and osteoporosis as well as disorders associated with disruption of the circadian rhythm. Our recent development of multiple synthetic RORα ligands offers the opportunity to modulate the metabolic pathways regulated by RORα and the ability to validate RORα as a drug target using a chemical biology approach [5], [6], [7], [8], [9].
